# In-vivo X-ray Dark-Field Chest Radiography of a Pig

**DOI:** 10.1038/s41598-017-05101-w

**Published:** 2017-07-06

**Authors:** Lukas B. Gromann, Fabio De Marco, Konstantin Willer, Peter B. Noël, Kai Scherer, Bernhard Renger, Bernhard Gleich, Klaus Achterhold, Alexander A. Fingerle, Daniela Muenzel, Sigrid Auweter, Katharina Hellbach, Maximilian Reiser, Andrea Baehr, Michaela Dmochewitz, Tobias J. Schroeter, Frieder J. Koch, Pascal Meyer, Danays Kunka, Juergen Mohr, Andre Yaroshenko, Hanns-Ingo Maack, Thomas Pralow, Hendrik van der Heijden, Roland Proksa, Thomas Koehler, Nataly Wieberneit, Karsten Rindt, Ernst J. Rummeny, Franz Pfeiffer, Julia Herzen

**Affiliations:** 1 Chair of Biomedical Physics & Institute of Medical Engineering, Technical University of Munich, 85748 Garching, Germany; 2 Department of Diagnostic and Interventional Radiology, Klinikum rechts der Isar, Technical University of Munich, 81675 München, Germany; 3 Institute of Medical Engineering, Technical University of Munich, 85748 Garching, Germany; 4 Institute of Clinical Radiology, Ludwig-Maximilian-University Hospital Munich, 81377 Munich, Germany; 5 Institute of Molecular Animal Breeding and Biotechnology, Ludwig-Maximilian-University, 85764 Oberschleißheim, Germany; 6 Institute of Microstructure Technology, Karlsruhe Institute of Technology, 76344 Eggenstein-Leopoldshafen, Germany; 7 Philips Medical Systems DMC GmbH, 22335 Hamburg, Germany; 8 Philips GmbH Innovative Technologies, Research Laboratories, 22335 Hamburg, Germany; 9 Institute for Advanced Study, Technical University of Munich, 85748 Garching, Germany

## Abstract

X-ray chest radiography is an inexpensive and broadly available tool for initial assessment of the lung in clinical routine, but typically lacks diagnostic sensitivity for detection of pulmonary diseases in their early stages. Recent X-ray dark-field (XDF) imaging studies on mice have shown significant improvements in imaging-based lung diagnostics. Especially in the case of early diagnosis of chronic obstructive pulmonary disease (COPD), XDF imaging clearly outperforms conventional radiography. However, a translation of this technique towards the investigation of larger mammals and finally humans has not yet been achieved. In this letter, we present the first *in*-*vivo* XDF full-field chest radiographs (32 × 35 cm^2^) of a living pig, acquired with clinically compatible parameters (40 s scan time, approx. 80 µSv dose). For imaging, we developed a novel high-energy XDF system that overcomes the limitations of currently established setups. Our XDF radiographs yield sufficiently high image quality to enable radiographic evaluation of the lungs. We consider this a milestone in the bench-to-bedside translation of XDF imaging and expect XDF imaging to become an invaluable tool in clinical practice, both as a general chest X-ray modality and as a dedicated tool for high-risk patients affected by smoking, industrial work and indoor cooking.

## Introduction

The lung consists of several hundred million air-tissue interfaces (formed by alveoli walls) that provide sufficient gas exchange for breathing. As clinically used conventional attenuation-based radiography of the lung cannot resolve these microstructures, its diagnostic window is mostly restricted to indirect signs caused by late-stage pathologies. In contrast, XDF radiography^1^ is sensitive to the pulmonary micromorphology itself, as the aforementioned interfaces cause significant ultra-small-angle X-ray scattering and a corresponding XDF signal.

Recent small-animal studies demonstrated that XDF imaging enhances pulmonary diagnosis, e.g. for the early detection and staging of COPD^2–4^, pulmonary fibrosis^5^, pneumothoraces^6^ and neonatal lung injury associated with mechanical ventilation^7^. Furthermore, the assessment of pulmonary carcinoma, edema, as well as pneumonia may significantly benefit from XDF imaging. These pathologies are characterized by a destruction (as in the case of COPD) or densification (by fibrotic or tumorous tissue) of the natural alveolar structure as the disease progresses. The loss of air-tissue interfaces consequently results in a reduction of the XDF signal compared to the distinct signal of healthy lung tissue. Hence, variations in the XDF lung pattern can indicate pathological changes. The combination of XDF with conventional imaging can be used for differential diagnosis^8^.

In order to measure the XDF signal (of lungs and other specimens), different approaches have been developed^1, 9–15^. Among these, grating-based imaging (GBI) methods (i.e. grating interferometers^1, 10–13^ and coded apertures^14, 15^) are most promising for clinical applications, as they are compatible with conventional X-ray sources and detectors. Additionally, GBI has the advantage of simultaneously acquiring a conventional attenuation image alongside the XDF signal, providing the radiologists with both functional (XDF) and anatomical (attenuation) imaging by means of a single scan only.

In GBI, an arrangement of three gratings with periods in the range of micrometers is placed in the X-ray beam to create a fringe pattern on the detector. The contrast of the interference pattern is commonly referred to as the (interferometric) visibility of the system and the XDF signal is subsequently given by the reduction in visibility when a scattering specimen is introduced into the beam path.

XDF chest imaging has until now been limited to the investigation of small animals or *ex*-*vivo* samples. *In*-*vivo* imaging of larger mammals still poses substantial technical challenges: first, the entire thorax has to be covered, which demands a field-of-view (FOV) of at least 30 × 30 cm^2^. Second, to minimize motion artifacts, the full scan has to be conducted during a single (induced) breath stop, which should not exceed one minute due to animal care regulations. Current GBI systems offer a maximal FOV of about 10 × 10 cm^2^, contingent on the fabrication limitations of the gratings. One simple way to increase the effective FOV is to use multiple exposures and subsequently stitch the obtained images together, which results in excessively long scan times and unfavorable movement of the specimen^16^. To overcome this restriction and to increase the FOV, scanning approaches^17, 18^ and tiled gratings^19^ have been suggested. Apart from the large FOV, the images have to be acquired at parameters compliant with clinical standards. Finally, the images have to be acquired at clinically relevant energies (typically 60–150 kVp for chest radiography). While first applications of high-energy XDF have been carried out as proof-of-principle investigations^20, 21^, none of the proposed systems satisfied all of the above requirements at once.

**Figure 1:**
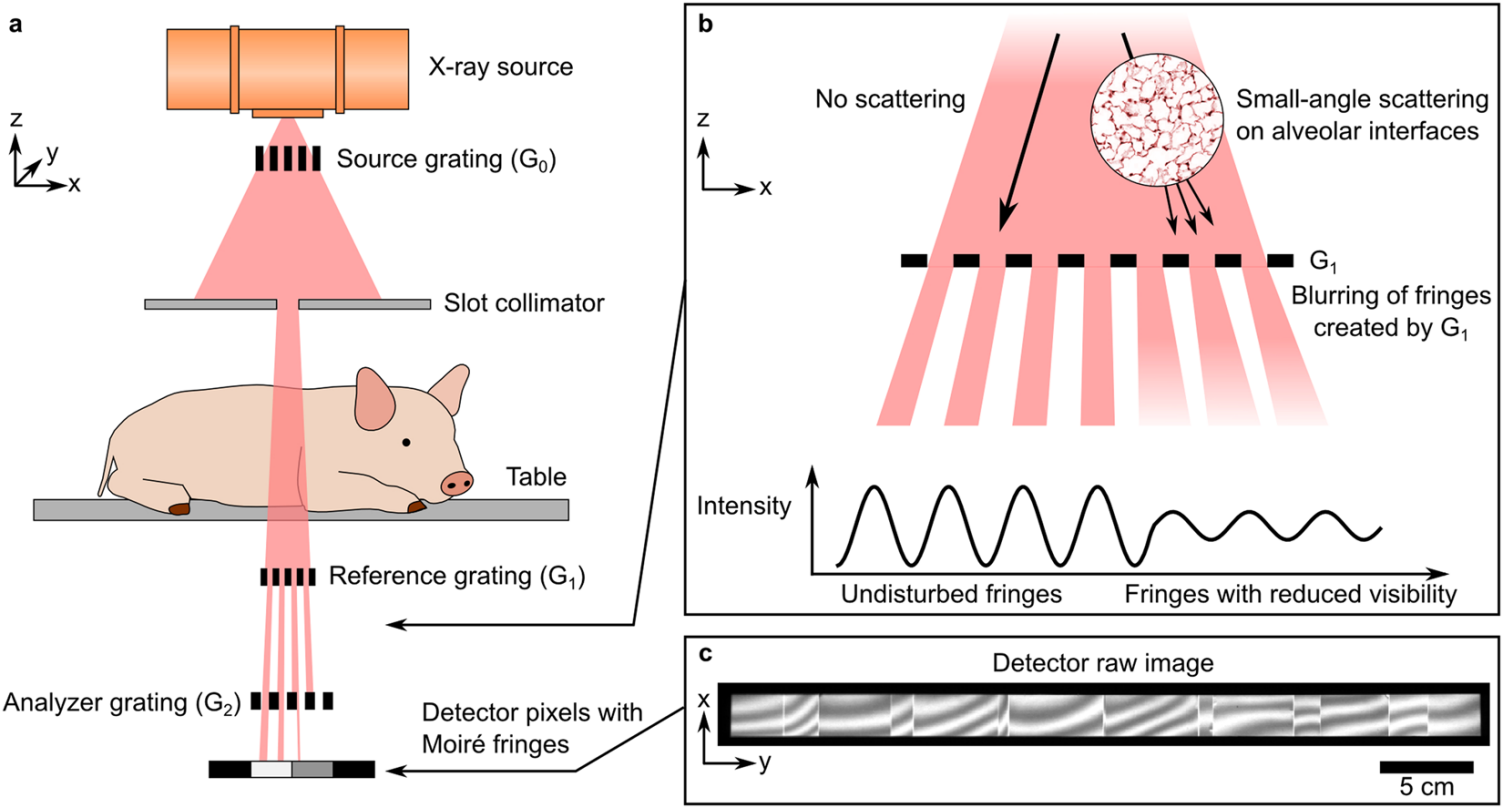
Full-field X-ray dark-field (XDF) chest radiography scanner. (**a**) Schematic of the prototype. A coarse array of Moiré fringes serves as a reference pattern created by a slight mismatch between the G_1_ and G_2_ grating orientation. The anesthetized pig is placed on a sample bed and scanned by a continuous movement. The influence of the sample on the Moiré fringe is used to calculate the XDF images. (**b**) In case of the lung, millions of micron-sized alveoli (more precisely their air-tissue interfaces) scatter the X-rays, causing a blurring and subsequent decrease of the G1 fringe visibility. (**c**) Raw detector image with the reference Moiré fringe pattern. Note that the vertical strikes arise from stitching together the borders of neighboring grating tiles and that the scale bar corresponds to the dimensions in the detector plane.

In this letter, we present the first XDF chest radiographs with a FOV of 32 × 35 cm^2^ of a living pig acquired with a novel high-energy XDF scanner (illustrated in Fig. 1a) in 40 seconds scan time.

Our system achieves high visibilities when operating at high X-ray energies (31% at 70 kVp) and is built in a compact system design. To achieve the necessary FOV, we combined a slot-scanning approach with linearly tiled gratings of about 40 × 2.5 cm^2^.

Porcine thorax anatomy is considered to closely resemble that of humans and pigs are therefore a widely accepted model for translational respiratory medicine^22^. Figure 1 shows how the anesthetized and mechanically ventilated animal was placed on a sample bed in the scanner. During the image acquisition process, ventilation was paused and a constant pressure of 11 mbar was applied to the lungs in order to simulate an intermediate depth of inspiration.

**Figure 2:**
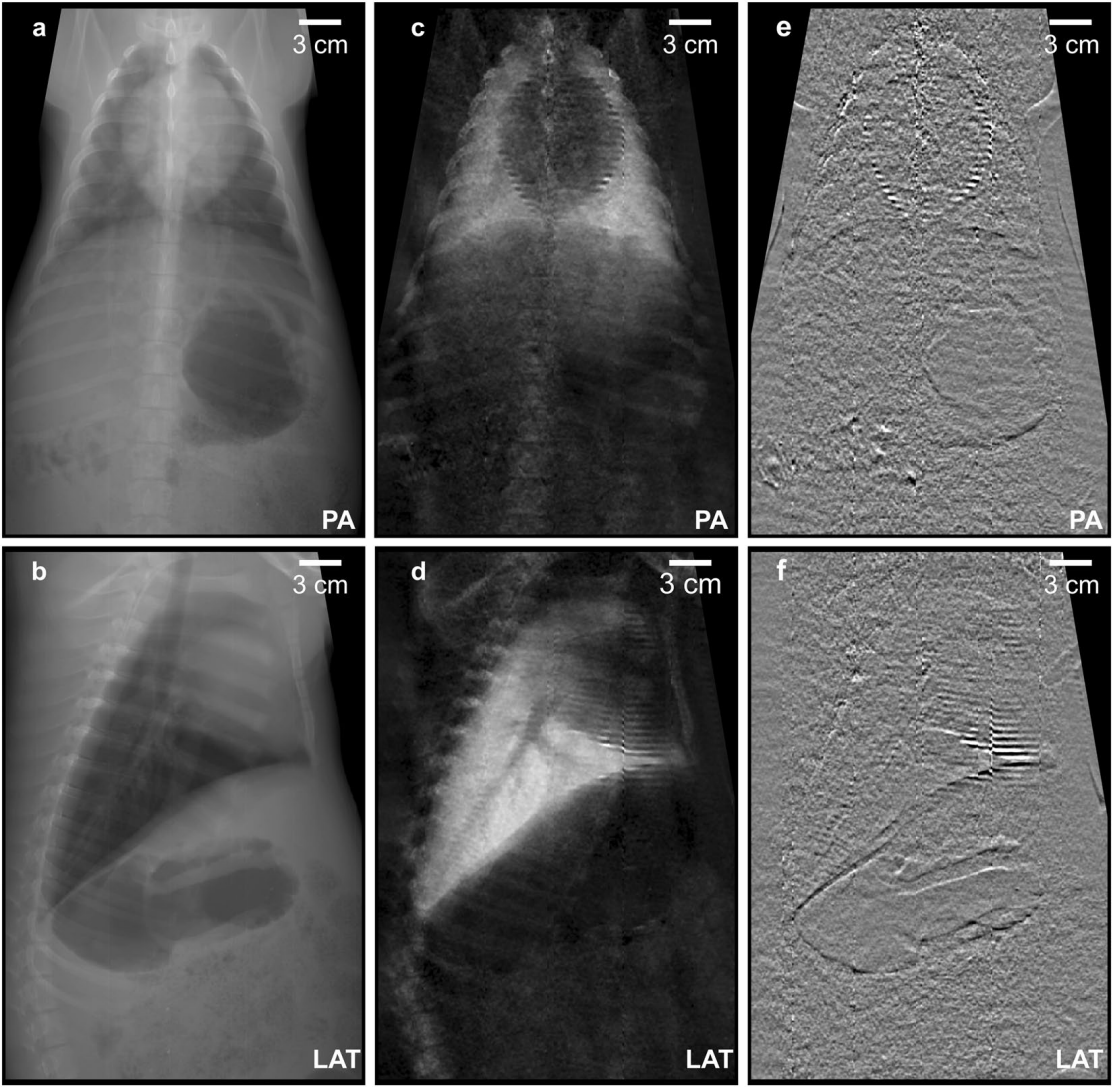
First *in*-*vivo* porcine multi-contrast chest radiographs. Attenuation (**a**,**b**), X-ray dark-field (**c**,**d**) and differential phase (**e**,**f**) chest radiographs of a healthy, living pig in posteroanterior (PA) (top row) and lateral (LAT) view (bottom row). Both scans were conducted using imaging parameters compliant with animal care, namely 40 seconds total scan time and a radiation dose of approximately 80 µSv. In particular the XDF radiographs (**c**,**d**) allow for an easy and unambiguous assessment of the pig lung, since overlying structures (e.g. fat) present only negligible scattering, and the XDF signal strength is correlated to the number of alveolar interfaces. Please note: images **a**–**d** are displayed as the neg. natural logarithm of relative transmission and visibility loss respectively.

Figure 2 illustrates as an example, results obtained for one animal in posterior-anterior view with 19.5 cm chest thickness (PA, Fig. 2 top row), and in lateral view (lying on its right side) with 16 cm lateral chest thickness (LAT, Fig. 2 bottom row), respectively. Both exposures (PA and LAT) were conducted with the same dose area product (DAP) of 0.5 Gy*cm^2^, resulting in an effective dose of approximately 80 µSv for the PA exposure. This value is compatible with conventional chest radiographs (20 µSv for PA examinations^23, 24^) and equals around ten days of natural background radiation^23^.

When comparing XDF (Fig. 2c,d) with the conventional radiographs (Fig. 2a,b), the diagnostic benefit of scatter-sensitive imaging in lung diagnostics becomes apparent: the XDF signal allows functional assessment of the pig lung, since the associated scattering signal is dominant in the thorax region. At the same time, the overlying and surrounding structures (e.g. fat, muscles and bones) hardly exhibit any scattering and therefore appear “dark-field transparent”. Thus they do not compromise the assessment and delineation of the lung, as is the case in attenuation radiographs. Note that the presented radiographs have sufficient quality to guarantee a meaningful radiographic assessment of the lung: the XDF signal exhibits a homogenous distribution/pattern throughout the lung, as expected in healthy animals.

**Figure 3:**
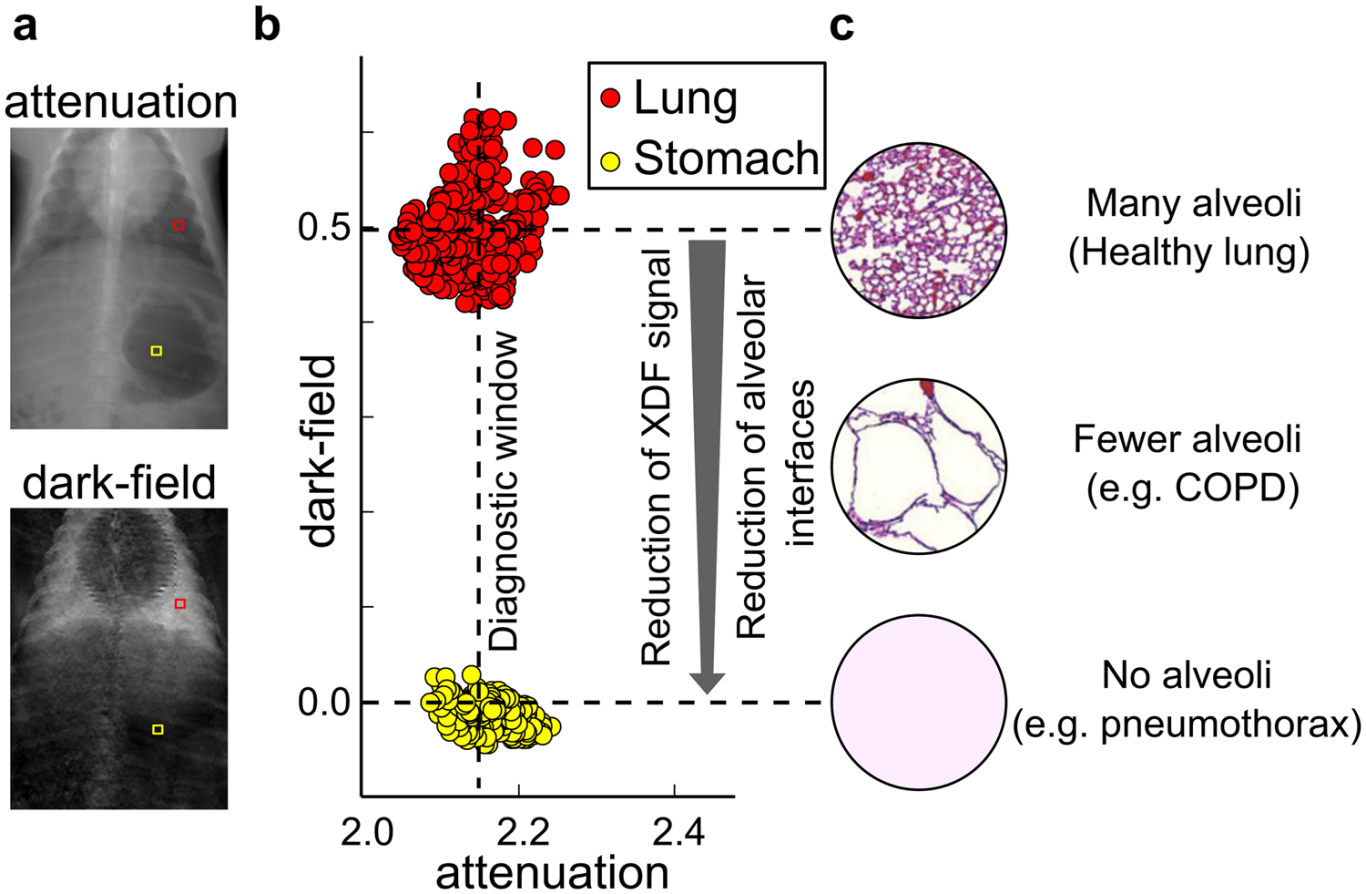
Potential of XDF imaging. (**a**) Two regions of interest with similar attenuation signals but different XDF behavior showcase the diagnostic potential of XDF imaging. (**b**) Scatterplot comparing healthy lung tissue (red) with intact alveolar interfaces and a strong XDF signal vs. the air-filled stomach (yellow) with no internal microstructure, and thus a small XDF value. As the XDF signal strength is directly correlated to the number of alveolar interfaces, a loss of the latter due to respiratory diseases, as indicated in the example of histopathological slices in (**c**), can be diagnosed even if the attenuation signal remains unaltered. The diagnostic window ranges up to the point where no alveoli are left, which is the case e.g. in a pneumothorax. For this extreme case, the air-filled stomach is considered only as a demonstrative model here.

In order to demonstrate the potential of the complementarity between XDF imaging and conventional radiography, Fig. 3 shows two regions of interest with similar attenuation but different XDF characteristics: healthy lung tissue with a large number of alveolar interfaces yielding a strong XDF signal versus the air-filled stomach with no inherent microstructure and thus no XDF contrast. Experiments with mice^3–7^ proved that the XDF signal strength is directly correlated to the number of intact alveolar interfaces. Therefore, any pathology associated with their loss will continuously reduce the XDF signal up to the point where no interfaces are left at all. This opens up a new diagnostic window to detect early stages of respiratory diseases, which typically appear radiolucent on the attenuation radiograph. The extreme case, in which lung tissue/alveolar interfaces are completely displaced by air, would e.g. occur in a pneumothorax or bulla, for which the air-filled stomach is only considered as an educational and demonstrative model here. The scatterplot in Fig. 3b further demonstrates the feasibility (in the case of pigs) of discriminating tissues with similar attenuation properties based on their XDF signal intensities.

Besides the attenuation and XDF images, GBI also provides differential-phase images^10, 11, 13^. These are shown in Fig. 2e,f. Note, however, that our system was designed to accommodate the strong signal expected for pulmonary XDF imaging, and hence only yields a moderate phase sensitivity^25^. Therefore, the differential-phase images of the *in*-*vivo* pig in our experiments provide little additional information (compare Fig. 2e,f).

Our results represent a breakthrough in the translation of XDF imaging to clinical applications. As neither simulations nor *ex*-*vivo* studies could prove that a translation of the technique from mice to humans is feasible, we used a living pig as a realistic model. The results presented address the major challenges of chest XDF imaging, namely: achieving a large FOV, high visibility at clinically compatible X-ray energies, a short acquisition time, and a clinically acceptable radiation dose.

Our findings with the study of a healthy pig support the assumption that the XDF signal seen in the pig lung originates from the same morphological structure, namely: the air-tissue interfaces of the alveoli, as shown in various mice models. In the next step, follow-up studies are needed to quantify the diagnostic sensitivity and specificity of XDF imaging in further large animal models and/or clinical trials.

XDF imaging has the potential to close the diagnostic gap between microscopic but invasive histopathology and conventional macroscopic X-ray imaging. We believe that XDF imaging can be established as a functional imaging tool in clinical practice. As lung diseases like COPD belong to the leading causes of mortality and morbidity in modern society^26^, any improvements in establishing an early diagnosis for these diseases will benefit a large number of people. Finally, our results open the way for other XDF imaging applications that require large objects to be scanned in a relatively short time. This could include other medical applications such as improved osteoporosis fracture risk assessment^27^ and novel XDF micro-bubble contrast agents^28^, as well as non-medical fields like airport security and material or food science.

## Methods

### Dark-field chest scanner

The core part of the scanner consists of three gratings (G_0_, G_1_, G_2_). The source grating G_0_ exhibits an active area of 5.0 × 2.5 cm^2^, shaping the extended focal spot into an array of fine slit sources, and preventing blurring of the fringe pattern. The reference grating G_1_ and analyzer grating G_2_ both have an area of 40 × 2.5 cm^2^, obtained by tiling 8 smaller sub-tiles of 5.0 × 2.5 cm^2^. Grating tiles were assembled under a microscope, with stitching gaps smaller than one pixel column. Coarse Moiré fringes for imaging were intentionally created on the detector by introducing a small periodic mismatch between the G_1_ and the analyzer grating G_2_ (see Fig. 1c). The system has a source-to-detector distance of 2.0 m. The X-ray source is a *Philips SRO 1750 ROT 360* (*Philips Medical Systems*, *Hamburg*, *Germany*) operated at 70 kVp and the detector is a first-generation *Pixium RF 4343* (*Trixell*, *Moirans*, *France*). To achieve a high readout rate (currently 12 Hz), the detector is operated in a fluoroscopy, 3 × 3-binning mode with a resulting pixel size of 444 µm.

### Moiré fringe visibility

The scanner achieves a visibility of 31 ± 4% over the full FOV with a clinical 70 kVp tungsten spectrum. Nevertheless, the visibility still changes when measuring a highly attenuating sample like a thorax due to an unavoidable energy dependency of the grating interferometer. Consequently, to account for beam hardening in the measured XDF signal, calibration is conducted prior to the measurements^29, 30^ with different equivalent attenuators made of polyoxymethylene (POM).

### Image acquisition

Image acquisition and data processing rely on a Moiré fringe scanning approach^17, 18^ in which there is an intrinsic movement in the x-direction (Fig. 1) between the gratings and the sample. This scanning movement samples every point in the object with different phases of the Moiré pattern. Finally, one scan contains a “pseudo stepping curve” of twenty-five pulsed exposures per image pixel, each acquired with an exposure time of 20 ms and tube current of 340 mA. These short x-ray pulses allow for a continuous motor movement while preserving quasi-static images. By fitting the data of scans with and without a sample, the three imaging modalities (attenuation, dark-field and differential phase) can be extracted. All radiographic images presented here have an effective FOV of 32 × 35 cm^2^ with an effective pixel size of approx. 360 µm in the sample plane. The scan time of 40 seconds is sufficiently short to suspend breathing in mechanically ventilated animals – although it is still challenging for patients with impaired lung function. In the current configuration, the X-ray tube power and the detector readout speed limit the minimum scan time. In addition to the pig experiments, we imaged an arrangement of fruits to demonstrate the system performance across the full FOV (extended data Fig. 1). All attenuation and XDF images are displayed as the negative natural logarithm of the relative transmission and visibility loss respectively, so that they are linear with respect to the sample thickness. The differential phase images are shown in radians.

### Motion artifacts

Our experiments showed that the shape of the Moiré fringe pattern (see Fig. 1c) determines the strength of the image artifacts introduced by the beating heart. The fringe scanning procedure assumes that all intensity modulations during a scan are the result of “scanning” through the phase of the Moiré fringe. However, the beating heart introduces an additional intensity fluctuation, especially at the heart-lung boundary, resulting in data inconsistencies. To reduce these artifacts, a larger fringe period seems to be beneficial (with the upper limit being determined by the requirement that one full period must still be contained in the grating slot to employ the fringe-scanning algorithm). As the remaining artifacts (see Fig. 2) are limited to the heart-lung boundary, they should not degrade the diagnostic quality of the images.

### Dose calculation

Assuming an adequate similarity between porcine and human thoraxes for PA projections, we used the conversion factor K given by Wall *et al*.^31^ to calculate a rough estimate of the effective radiation dose (ED) from the measured dose area product (DAP) via: ED[mSv] = K * DAP[Gy*cm^2^].

The incident air kerma at the table was measured by a *PTW NOMEX* dosimeter (*PTW*, *Freiburg*, *Germany*) and the illuminated area was estimated from the FOV, resulting in a DAP of 0.5 Gy*cm^2^. With K = 0.16 [mSv/ (Gy*cm^2^)] (ICRP 103 at 120 kVp) an ED of 80 µSv was calculated for the PA exposure. The conversion factor for a tube voltage of 70 kVp will be lower and consequently our reported effective dose values are slightly higher than the actual dose.

### Animals

One German Landrace Hybrid pig (wildtype, Institute of Molecular Animal Breeding and Biotechnology, Ludwig Maximilian University Munich breeding facility; *n* = 1; male; weight = 23 kg; age 3 months) was sedated by intramuscular application of Ketamine (Ursotamin®, Serumwerk Bernburg, Germany, 20 mg/kg) and Azaperone (Stresnil®, Elanco Animal Health, Bad Homburg, Germany 2 mg/kg). Anesthesia was continued by intravenous injection with Propofol (Propofol 2%, MCT Fresenius, Fresenius Kabi, Langenhagen, Germany) using a syringe pump (Injectomat® MC Agilia, Fresenius Kabi, Langenhagen, Germany) with dose adjusted to effect. The animal was kept under automated ventilation throughout the experiment. For imaging, ventilation was paused for the duration of the scan (max. 60 sec at a time) with a constant pressure of approx. 11 mbar in the airways. Heart rate and oxygenation were monitored continuously. All animal procedures were performed with permission of the local regulatory authority, Regierung von Oberbayern (ROB), Sachgebiet 54, 80534 Munich, approval number AZ 55.2-1-54-2532-61-2015. The application was reviewed by the ethics committee according to §15 TSchG German Animal Welfare Law. All experiments were performed in accordance with relevant guidelines and regulations. To terminate the experiment, the animal was euthanized under anesthesia by intravenous injection of T61® (Intervet GmbH, Unterschleissheim, Germany) according to the manufacturer’s instructions. No randomization or blinding was performed.﻿

### Data availability

The datasets generated during and/or analyzed during the current study are available from the corresponding author on reasonable request.

## Electronic supplementary material

Supplementary Figure 1
