# Supplementary material for: In-vivo X-ray Dark-Field Chest Radiography of a Pig
Source: Sci Rep. 2017 Jul 6;7:4807. doi: 10.1038/s41598-017-05101-w (PMC5500502; doi:10.1038/s41598-017-05101-w)
Supplement: Supplementary file 1 — Supplementary Figure 1 [file 41598_2017_5101_MOESM1_ESM.pdf]

In-vivo X-ray Dark-Field Chest Radiography of a Pig

Authors: Lukas B. Gromann[1]*, Fabio De Marco*[1], Konstantin Willer[1], Peter B. Noël[1,2], Kai Scherer[1], Bernhard Renger[2], Bernhard Gleich[3], Klaus Achterhold[1], Alexander A. Fingerle[1,2], Daniela Muenzel[1,2], Sigrid Auweter[4], Katharina Hellbach[4], Maximilian Reiser[4], Andrea Baehr[5], Michaela Dmochewitz[5], Tobias J. Schroeter[6], Frieder J. Koch[6], Pascal Meyer[6], Danays Kunka[6], Juergen Mohr[6], Andre Yaroshenko[1,7], Hanns-Ingo Maack[7], Thomas Pralow[7], Hendrik van der Heijden[7], Roland Proksa[8], Thomas Koehler[8,9], Nataly Wieberneit[7], Karsten Rindt[7], Ernst J. Rummeny[2], Franz Pfeiffer[1,2,9], Julia Herzen[1]

[1]Chair of Biomedical Physics & Institute of Medical Engineering, Technical University of Munich, 85748 Garching, Germany.
[2]Department of Diagnostic and Interventional Radiology, Klinikum rechts der Isar, Technical University of Munich, 81675 München, Germany.
[3]Institute of Medical Engineering, Technical University of Munich, 85748 Garching, Germany.
[4]Institute of Clinical Radiology, Ludwig-Maximilian-University Hospital Munich, 81377 Munich.
[5]Institute of Molecular Animal Breeding and Biotechnology, Ludwig-Maximilian-University, 85764 Oberschleißheim.
[6]Institute of Microstructure Technology, Karlsruhe Institute of Technology, 76344 Eggenstein-Leopoldshafen, Germany.
[7]Philips Medical Systems DMC GmbH, 22335 Hamburg, Germany.
[8]Philips GmbH Innovative Technologies, Research Laboratories, 22335 Hamburg, Germany.
[9]Institute for Advanced Study, Technical University of Munich, 85748 Garching, Germany.
*These authors contributed equally to this work

Extended Data:

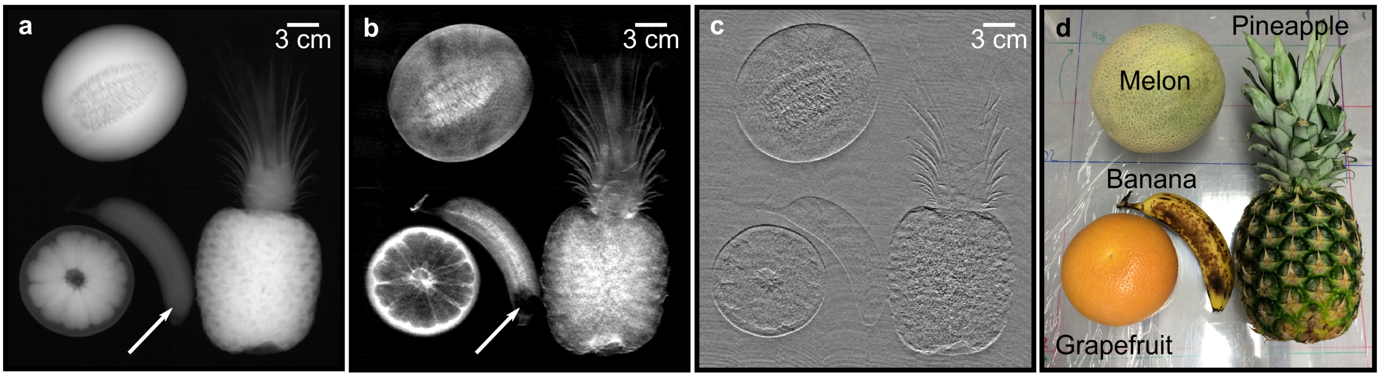

Extended data figure 1: Full-field multi-contrast X-ray radiographs of a fruit arrangement. **(a)** Full-field X-ray attenuation, **(b)** dark-field, **(c)** differential-phase radiographs, and **(d)** photograph of a fruit arrangement used as a test phantom to visualize the performance of the scanner across the entire FOV (32 x 35 cm^2). Note that the XDF channel provides complementary information not accessible from the conventional attenuation image: in the lower tip of the banana the fibrous cellular structure was destroyed by squeezing it prior to imaging, detectable in the XDF image as a loss in scattering signal (white arrow). The differential-phase mainly shows the edges of the fruit.
